# BIRTH WEIGHT, WEIGHT GAIN, AND OBESITY AMONG CHILDREN IN URUGUAY: A PROSPECTIVE STUDY SINCE BIRTH

**DOI:** 10.1590/1984-0462/2021/39/2019088

**Published:** 2020-08-26

**Authors:** Isabel Pereyra, Andrea Gómez, Karina Jaramillo, Augusto Ferreira

**Affiliations:** a Universidad Católica del Uruguay, Montevideo, Uruguay.; b University of Chile, Santiago, Chile.

**Keywords:** Birth weight, Body mass index, Body weight changes, Obesity, Longitudinal studies, Peso ao nascer, Índice de massa corporal, Alterações do peso corporal, Obesidade, Estudos longitudinais

## Abstract

**Objective:**

To examine the effect of birth weight and subsequent weight gain on children being overweight and obese in serial assessments of Uruguayan children living at urban areas.

**Methods:**

We used secondary data of pediatric anthropometric measurements and health and socioeconomic characteristics of families that were included in a longitudinal and prospective nationally representative survey (“*Encuesta de Nutrición, Desarrollo Infantil y Salud*”). The associations of conditional weight gain, being overweight and obesity were tested through correlation coefficients. Multivariate binary logistic regression models were performed to calculate the effect of birth weight on childhood obesity and were adjusted for covariates.

**Results:**

For macrosomic babies, there was an increase in the prevalence of overweight and obesity in 70% compared with non-macrosomic babies, when we adjusted for sex, exclusive breastfeeding duration, and household income. The correlation between weight gain and the body mass index for age indicated that the greatest (positive) difference in Z score between measurements increased the obesity levels.

**Conclusions:**

Our findings suggest that ensuring optimal birth weight and monitoring and controlling posterior weight gain represent the first steps toward primary prevention of childhood obesity.

## RESUMO

**Objetivo:**

Analisar o efeito do peso ao nascer e do ganho ponderal subsequente em crianças com sobrepeso e obesidade com base em avaliações consecutivas de crianças uruguaias vivendo em áreas urbanas.

**Métodos:**

Foram utilizados dados secundários de medidas antropométricas pediátricas, além de características de saúde e socioeconômicas de famílias incluídas em um inquérito prospectivo e longitudinal de representatividade nacional (“*Encuesta de Nutrición, Desarrollo Infantil y Salud*”). As associações entre ganho ponderal condicional, sobrepeso e obesidade foram testadas por meio de coeficientes de correlação. Modelos de regressão logística binária multivariada foram construídos para calcular o efeito do peso ao nascer sobre a obesidade infantil e ajustados por covariáveis.

**Resultados:**

Bebês macrossômicos tiveram um aumento de 70% na prevalência de sobrepeso e obesidade em comparação a bebês não-macrossômicos, quando ajustado por sexo, duração do aleitamento materno exclusivo e renda familiar. A correlação entre ganho ponderal e índice de massa corporal para idade mostrou que a maior diferença (positiva) de escore z entre as medições aumentou os níveis de obesidade.

**Conclusões:**

Os achados deste estudo sugerem que garantir o peso ideal ao nascer e monitorar e controlar o ganho ponderal subsequente são os primeiros passos para a prevenção primária da obesidade infantil.

## INTRODUCTION

Changes in intrauterine and early postnatal growth (critical periods of human development) may have long-term implications for later health.^1^ ^,^ ^2^ Newborn size and postnatal progression rates are important determinants of human perinatal survival, which are influenced by the mother’s genetic, environmental and placental factors.

Obesity is the primary nutritional concern in childhood, and this is increasing in most regions of the world. This childhood obesity rise is alarming because, in addition of being a disease in itself, it is one of the main risk factors for Chronic Non-Communicable Diseases.

Previous epidemiological studies have identified factors of early life stages that favor the development of obesity in children, such as: maternal weight, gestational diabetes, birth-weight, feeding with different milk formulas, early introduction of solid foods, accelerated weight gain patterns in the first months of life, maternal smoking during pregnancy, low educational level of parents, high birth weight, family obesity, excessive television screen time, and electronic games.^3^ ^,^ ^4^

Birth weight and accelerated weight gain during the first months of life have been previously reported as leading to childhood obesity.^5^ Studies have been published and suggest a “J” or “U” relationship, with an increased risk of obesity in extremes of birth weight.^6^ Being born with a weight above the 90th percentile or large for gestational age (LGA) would indicate an adverse intrauterine environment.^7^ Macrosomic (birth weight greater than or equal to 4000 g) and LGA newborns have a greater risk of developing obesity during school age. In children with a normal weight at birth, weight gain during the first six years of life is an important risk factor for obesity, especially when it occurs during the first years of preschool period.^8^ Macrosomia is a relatively new phenomenon in developing countries, and its impacts on obesity have not been well studied yet.

In 2011, in Uruguay, obesity in children under two years was 9.5%.^9^ The results of the Brazilian First National Survey of Health, Nutrition and Child Development (ENDIS), published in 2015, highlighted a prevalence of overweight-obese of 10.5%.^10^

The identification of risk factors is important for prevention. The concept of a life cycle, in which each period is dependent on the previous ones, provides a framework for Public Health and shows that prevention must begin before risk factors are developed. Therefore, it would be highly recommended that prevention of obesity began in early childhood.^11^ Longitudinal studies provide the advantage of being able to evaluate cumulative risk during a certain time interval.

This study aimed to examine the effect of birth weight and subsequent weight gain on childhood obesity in consecutive assessments of Uruguayan children living in urban areas throughout the national territory.

## METHOD

The “*Encuesta de Nutrición, Desarrollo Infantil y Salud*” *-* ENDIS (Survey of Childhood Nutrition, Development and Health) *-* is an ongoing longitudinal, prospective cohort study conducted since birth. The study examines: nutritional status, development, health, socioeconomic and demographic characterization, identification and access to social benefits, food safety, child feeding practices, parenting practices at home, women’s health and sexual and reproductive health, access to and use of health services (check-ups, immunization, use of supplements or medicines, access to contraceptive methods), home organization, parenting environment, and household income between infant populations in Uruguay.

The sample was randomized and comprised two selection phases. The first phase sample corresponds to the Continuous Household Survey, in which the design was randomized and stratified in two or three selection stages. Then, in the second phase, all households were selected and met the condition of having children younger than four years of age. This was because the number of households who followed this characteristic in the Continuous Household Survey was the minimum sufficient to obtain estimates with reasonable levels of accuracy and confidence for the different indicators and levels of disaggregation proposed for the study.^12^ ^,^ ^13^

**Figure 1:**
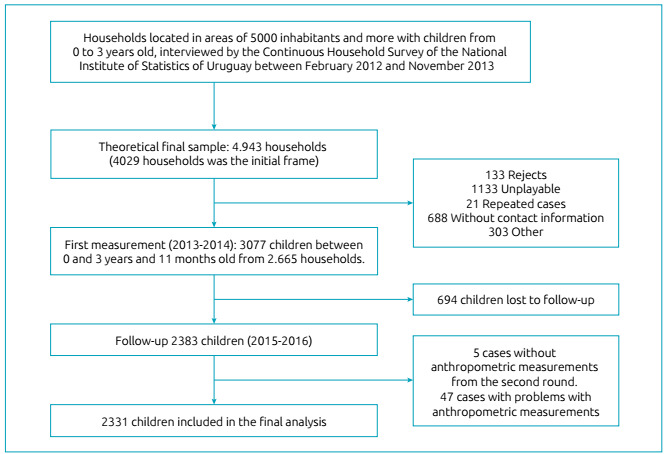
Study flow chart.

The ENDIS project has two measurements. One of them was from 2013‒2014 that followed 3,077 infants living in Uruguay, between birth and three years and 11 months. The other is more recent, from 2015‒2016, and there were 694 losses at follow-up (n=2383).^14^ Eligible infants lived in households who met the Continuous Household Survey. We studied only 2,383 because the other 694 did not have the second measurement (Figure 1).

The ENDIS project was approved by the Ethics Committee of the School of Medicine from the University of the Republic of Uruguay (Resolution no. 159 of the session from March 18, 2013 from the School of Medicine, file number 070153-000486-13). Informed consent was provided in writing according to the requirements of the Declaration of Helsinki (1995).

Birth size was noted from hospital records. The analysis of birth weight was used as a dichotomous qualitative variable measured as a macrosomic or non macrosomic newborn. An infant weighting equal to or greater than 4000 g at birth was considered macrosomic.

We studied the differences in the Z score of body mass index for age (BMI-for-age) and sex and for weight for age (WA) and sex. The Z scores analyzed were Z0 measured at birth, Z1 at age t1 (years: 2013‒2014), and Z2 at age t2 (years: 2015‒2016). To adjust for regression to the mean, the Z score on the second occasion was compared with what would be predicted from the first occasion. We started with the R computation of the correlation and then adjusted as follows: z2=r×z1. This adjustment was necessary because it is widely understood that the second is a conditional on the previous measurement.^15^ ^,^ ^16^

Weight and height were surveyed on the survey day, with double measurement of each parameter. We calculated Z scores for BMI-for-age and sex. Obese was defined as Z score≥3SD and overweight (OW) Z score≥2SD.^17^

Covariates were sex, household income (low, middle and high), maternal education (primary, secondary incomplete, secondary complete and university), breastfeeding duration (measured as months), and maternal age at birth.

The *Statistical Package for the Social Sciences* (SPSS for Windows 22.0.; SPSS Inc, Chicago, IL) was used for statistical analyses. Descriptive analysis and multivariable regression were applied for calculation. *Odds Ratios* (ORs) had 95% confidence intervals (95%CIs) and were adjusted for sex, maternal education, household income, delivery and breastfeeding duration. Logistic regression models were used to calculate the OR since it allows calculating the risk adjusted for confusing variables (bias control). This type of mathematical models does not allow calculating adjusted RR.^18^ In addition, it is based on the comparability strategy of results through the adjusted OR report with other studies. The values of quantitative variables are reported as means SDs unless otherwise stated. Relationships between quantitative variables were assessed by correlation and partial correlation coefficients. The independent associations between conditional weight gain during childhood and birth weight were tested by regression analysis. The associations of conditional weight gain and OW and obesity were tested by correlation coefficients. When obesity (OW and obesity) was modeled as the outcome variable, weight gain was excluded as an exposure variable, because weight gain is one of the components of BMI-for-age (outcome variable). Model building was performed firstly by introducing covariates one by one and finally by testing the interaction between macrosomic and covariates.

All data were processed in the SPSS except for BMI-for-age and WA, which were calculated using Anthro Plus software OMS (version 1.0.4; World Health Organization; Geneva, Switzerland).

## RESULTS

3,077 children aged between zero and three years and 11 months participated in the first measurement. In the second measurement, of the 3,077 children surveyed in the first round, 2,383 were interviewed, which signifies a 77% response rate. Five cases that did not present data in any of the anthropometric measurements were excluded from the second-round analyses. In this study, univariate analysis was used for the total population and bivariate and multivariate analyzes were carried out upon 2,378 cases that had both measurements. Prior to the nutritional analysis, the data were observed in terms of completeness and value of the measurements. After this first analysis, 47 cases were excluded due to problems with anthropometric measurements, providing a total of 2,331.

The studied children’s characteristics are reflected in Table 1. The average age was 24.8 and 51.3 months, in the first and second rounds, respectively. 52% of the sample was male. Regarding the characteristics related to intrauterine growth, the mean birth weight found was 3277.3 g. 7.3% of the children were born with a weight equal to or greater than 4000 g, which is known as macrosomia - a risk factor for children’s health. 10.7% of the children were born prematurely. The survey indicates that exclusive breastfeeding lasted an average of 5.2 months. The average Z score of BMI-for-age was 0.69 in the first round and 0.73 in the second. 12% of the population were found to be OW and obese in the first round and in the second, this increased by 1% (Table 1).

**Table 1:** Characteristics of children studied on each measurement.

	Measurement 1 (n=3077) %		Measurement 2 (n=2378) %	
Sex				
Boys	51.9			
Girls	48.1			
Educational center attendance				
Yes	85.1		79.9	
Maternal education				
Primary school or no educational level			16.9	
Incomplete high school			32.6	
High school			23.7	
University			26.8	
Macrosomic	7.3			
Obesity				
n=2822 (1^st^ measurement)	12.0			
n=2326 (2^nd^ measurement)			13.2	
	Mean	SD	Mean	SD
Birth weight (g) n=2,977	3277.3	568.9		
Gestational age (weeks)	38.7	2.1		
Age (months)	24.8	10.9	51.3	11.1
Exclusive breastfeeding (months) n=2,648	5.2	3.0		

Table 2 shows the results of bivariate analyzes between excessive intrauterine growth (manifested by macrosomia) and obesity in each of the rounds. The prevalence of OW and obesity was always higher among children who were born macrosomic, and this increased further in the second round. No association was found between the two variables in the first round, according to the chi-square analysis. But in the second-round, children who had been born with macrosomia were more likely to be classified as obese in comparison to those who were born with lower birth weight. We estimated the RR for the development of obesity related to having been born macrosomic, and we found a RR=1.01 (95%CI 0.96‒1.07) in the first round. The RR for the second round was 1.10 (95%CI 1.02‒1.20).

**Table 2:** Macrosomic deliveries and overweight and obesity.

	Overweight and obesity	
	1^st^ measurement **(n=2731)**	2^nd^ measurement (n=2258)
Macrosomic deliveries	13.3%	20.9%*
Non-macrosomic deliveries	12.2%	12.8%

*p=0.004.

**Figure 2:**
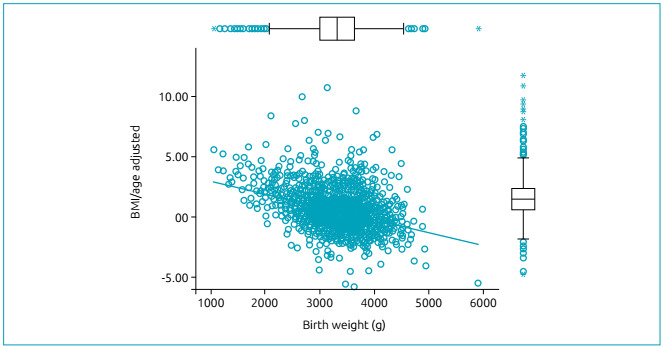
Conditional weight gain, according to birth weight.

The conditional weight gain analysis (from the difference in the Z score for BMI-for-age from birth to second measurement) and birth weight, using linear regression, determined that those born with lower weight were more likely to be categorized with greater weight gain (Figure 2).

According to these results, linear regression analysis allowed us to estimate that weight gain decreases with each point of the Z score for every kilo measured at birthweight in grams (Coefficient=-0.001, p<0.001). We also analyzed the adjusted weight gain for the WA index, but we did not find a significant association with birth weight in grams (p=0.968).

The analyzed conditional weight gain in the total measurement period (birth to second measurement) was associated with the Z score of the Weight-for-age and BMI-for-age. According to these results, the postnatal weight gain constitutes an important determinant of the Z score for the Weight-for-age and the BMI-for-age (Pearson’s correlation coefficient - R=0.97, p<0.001, R=0.81, p<0.001, respectively). The correlation between weight gain and BMI-for-age indicates that the greater (positive) difference in the Z score between measurements consequently increases obesity.

Weight gain in the Z score differences for BMI-for-age and obesity presence was analyzed by comparing the mean weight increases of the groups with and without OW and obesity. For this, the *t*-test was applied, resulting in both groups with a significant difference (p<0.001). The average weight gain for obese children was a Z score difference of 2.82, while for non-obese children, it was of 0.22.

To observe the effect of possible confounding variables, multivariate binary logistic regression models were performed. For OW and obese in the second round, direct associations were found with macrosomia and inverses with duration of exclusive breastfeeding in months and household income (measured in terciles), as seen in Table 3. The highest prevalence of risk (OW and obese) was found in, regarding macrosomia, delivery by cesarean, male sex and higher income households. Educational condition and maternal age did not show values with statistically significant differences for childhood obesity. The breastfeeding duration was not significant in the logistic regression analysis; however, the direction of the association was always the same: the longer the duration, the lower the BMI-for-age. The absence of association could be due to the number of cases without data in this variable (492 cases).

**Table 3:** Multivariate analysis of factors associated with overweight and obesity in the second measurement (n=2114).

	unadjusted RR (95%CI)
Macrosomia	1.10 (1.02-1.20)
	adjusted OR (95%CI)
Macrosomia	1.74 (1.10-2.76)
Sex	1.40 (1.06-1.83)
Socioeconomic status	0.65 (0.45-0.95)
Exclusive breastfeeding (months)	0.97 (0.92-1.02)
Delivery	1.43 (1.09-1.89)

## DISCUSSION

The results strongly support the contention that high birth weight and weight gain are significant risk factors for childhood obesity. Based on our results, children in the upper tertile of household income had a greater risk of obesity as per anthropometric measurements. In this follow-up study, males showed pieces of evidence of greater risk of obesity compared with females.

The strength of our study is the large sample size and data obtained by use of a standardized questionnaire across the country. To the best of our knowledge, this ﬁrst panel study focuses on fetal macrosomia and obesity in Uruguay. Our findings show that size at birth in this cohort was representative of the national data from the Brazilian Department of Health.^19^ The male:female ratio of 1.1:1 in our material was comparable to other studies.^20^ The ratio of macrosomic babies was high in this population, 7.3%, which is similar to other Latin-American countries, such as Argentina, Cuba, and Peru. Data from other countries are rare, but recent data from Brazil, Ecuador, Mexico and Nicaragua show lower prevalence, whereas it is higher in Paraguay.^20^

It is not known why Uruguayan babies are born heavier. Most studies around the world show that births of macrosomic babies are becoming more frequent. This tendency is not clear in Uruguay. Data from such country show that in 1999 the ratio of births of babies weighing 4000 g or greater was 6.6%, but by 2011 this had decreased to 6.1%.^9^ More recently, in the last two years, close to 8% of newborns were macrosomic.^19^ Over the past few decades, the rate of this disorder has increased worldwide, which could be due to the increased prevalence of diabetes and obesity in women at a reproductive age.^21^ Macrosomia is associated with increased risks of adverse delivery outcomes.^22^ Babies with macrosomia have an increased risk of birth trauma, asphyxia, and meconium aspiration, and their mothers have a high risk of abnormal hemorrhage, uterine atony, and prolonged labour.^23^

 In the previous decades, the prevalence of being OW and obese in children increased worldwide, and obesity is a growing concern. In developing countries, the transition from rural agrarian to urban economies has accelerated the appearance of obesity. A wealth of clinical and epidemiological evidence has linked obesity to a broad spectrum of cardiovascular diseases (CVD). The rise in obesity, thus, portends a worldwide increase in those chronic conditions associated with obesity and CVD, most importantly, coronary heart disease, heart failure, hypertension, stroke, atrial fibrillation, and sudden cardiac death.^24^ Excessive fat in childhood is a risk factor for later adult disease and is associated with impaired health during childhood itself, including increased risk of hypertension, insulin resistance, fatty liver disease, orthopedic dysfunction and psycho-social distress, which may continue untreated for many years. Once established, obesity in children (as in adults) is hard to reverse. Monitoring the preva­lence and risk factors of obesity, in order to plan services for the provision of care and to assess the impact of policy initiatives, is essential.^17^

Most researches about the relationship between prenatal exposures and later obesity have studied associations between birth weight and attained BMI. Birth weight can be easily measured, has reference norms, is part of the routine medical record, and may be available historically. Variation in weight at birth serves as a surrogate to reflect the underlying mechanisms influencing its growth.^25^

Childhood obesity has increased signiﬁcantly in recent decades in Uruguay.^26^ The difference in BMI-for-age between pediatric groups (macrosomic and non macrosomic) indicates that macrosomic babies tended to be heavier than babies within normal weight range at birth. This was more evident in the second measurement of the macrosomic babies, which is once more similar to other studies.^10^ For macrosomic babies, there was an increase in the prevalence of obesity in 70% compared with non-macrosomic babies. The current study showed that male gender and high family income are risks factors for OW and obesity. However, breastfeeding duration is a protective factor for obesity.

In a review of the literature, most studies showed a positive correlation between birth weight and childhood obesity.^1^ ^,^ ^25^ ^,^ ^27^ ^,^ ^28^ Many of these reports included epidemiologic studies with a large numbers of subjects.^25^ On the other hand, by adult BMI, several studies have examined the association with birth weight. Almost all of the studies have found direct associations, *i.e.* higher birth weight was associated with higher attained BMI.^25^ Some of the smaller studies have found no association, but none have found an inverse association.^25^

Monitoring growth during childhood is not as simple as it seems. Expressing weight gain as a centile or SD score requires knowledge of the mean and SD of weight gain between arbitrary ages, when published information on this is restricted mainly to time intervals of one, three, or six months. In this research, we used a sample of children aged between zero and four. Between these ages, the weight gain is quite different. Therefore, we adjusted weight gain with correlations because the second measurement is conditional to the previous.

Children who demonstrated more weight gain in the study had lower birth weight than other children. The reason why infants who had intrauterine growth restriction have greater postnatal weight gain is largely unknown, although greater food intake has been observed compared with other infants. Children who showed more weight gain in the study were heavier (weight-for-age) and fatter (BMI-for-age) at an average age of four in comparison with other children. The connection between weight gain during the first years of life and obesity is well described.^29^ Ong et al. have verified that children who displayed catch up in weight between zero and two years were heavier and taller than other children at 5. Furthermore, these children had greater BMI, percentage body fat, total fat mass, and central fat distributions, which are variables of childhood size, linked to metabolic markers for risk of disease in adulthood and are predictive of adulthood obesity. Thus, in contemporary, affluent societies the biological predisposition to catch-up growth conferred by intrauterine restraint may result in an acceleration of postnatal growth, which overshoots the genetic trajectory.^30^

Our study has several potential limitations. First, because the data were collected within 1‒2 years, seasonal and temporal variations could have introduced temporal bias. Furthermore, because the second measurement was available only in 2,383 infants, we only considered these infants in multivariate analysis. Finally, we had no information regarding breastfeeding of 492 infants, and thus its confounding and independent eﬀects are unclear. These potential limitations should be considered when the results are interpreted.

The worldwide epidemic of obesity continues unabated. Obesity is notoriously difficult to treat, and, thus, prevention is critical. The implication for current public health practice of this research is we need to understand that environmental factors in utero may influence lifelong health. A large number of epidemiological studies have demonstrated a direct relationship between birth weight and BMI attained in later life.^25^ Although data are limited by a lack of information on potential confounders, these associations seem robust. Future research on molecular genetics, intrauterine growth, growth trajectories after birth, and relationships of fat and lean mass will elucidate relationships between early life experiences and later body proportions. Prevention of obesity starting in childhood is critical and can have lifelong, perhaps multigenerational impacts.
